# Affinity depletion versus relative protein enrichment: a side-by-side comparison of two major strategies for increasing human cerebrospinal fluid proteome coverage

**DOI:** 10.1186/s12014-019-9229-1

**Published:** 2019-02-26

**Authors:** Eliska Jankovska, Marek Svitek, Karel Holada, Jiri Petrak

**Affiliations:** 1 BIOCEV, First Faculty of Medicine, Charles University, Prague, Czech Republic; 2 Department of Anesthesiology and Intensive Care, First Faculty of Medicine, Charles University, Prague, Czech Republic; 3 General University Hospital, Prague, Czech Republic; 4 Institute of Immunology and Microbiology, First Faculty of Medicine, Charles University, Prague, Czech Republic

**Keywords:** Cerebrospinal fluid, Depletion, CNS, Biomarkers, LC–MS/MS, Mass spectrometry

## Abstract

Cerebrospinal fluid (CSF) is in direct contact with the central nervous system. This makes human CSF an attractive source of potential biomarkers for neurologic diseases. Similarly to blood plasma, proteomic analysis of CSF is complicated by a high dynamic range of individual protein concentrations and by the presence of several highly abundant proteins. To deal with the abundant human CSF proteins, methods developed for blood plasma/serum are routinely used. Multiple affinity removal systems and protein enrichment of less abundant proteins using a combinatorial peptide ligand library are among the most frequent approaches. However, their relative impact on CSF proteome coverage has never been evaluated side-by-side in a single study. Therefore, we explored the effect of CSF depletion using MARS 14 cartridge and ProteoMiner ligand library on the number of CSF proteins identified in subsequent LC–MS/MS analysis. LC–MS/MS analysis of crude (non-treated) CSF provided roughly 500 identified proteins. Depletion of CSF by MARS 14 cartridge increased the number of identifications to nearly 800, while treatment of CSF using ProteoMiner enabled identification of 600 proteins. To explore the potential losses of CSF proteins during the depletion process, we also analyzed the “waste” fractions generated by both methods, i.e., proteins retained by the MARS 14 cartridge, and the molecules present in the flow-through fraction from ProteoMiner. More than 250 proteins were bound to MARS 14 cartridge, 100 of those were not identified in the corresponding depleted CSF. Similarly, analysis of the waste fraction in ProteoMiner workflow provided almost 70 unique proteins not found in the CSF depleted by the ligand library. Both depletion strategies significantly increased the number of identified CSF proteins compared to crude CSF. However, MARS 14 depletion provided a markedly higher number of identified proteins (773) compared to ProteoMiner (611). Further, we showed that CSF proteins are lost due to co-depletion (MARS 14) or exclusion (ProteoMiner) during the depletion process. This suggests that the routinely discarded “waste” fractions contain proteins of potential interest and should be included in CSF biomarker studies.

**Electronic supplementary material:**

The online version of this article (10.1186/s12014-019-9229-1) contains supplementary material, which is available to authorized users.

## Introduction

Proteomic analysis of human body fluids is a promising tool for the identification and detection of disease biomarkers. Human cerebrospinal fluid (CSF) is in direct contact with the central nervous system (CNS) and serves multiple functions including mechanic protection of the brain tissue, homeostasis maintenance, delivery of nutrients to the CNS, removal of waste and active regulation of CNS via hormones, neuropeptides, and other regulatory molecules. The composition of CSF thus reflects the physiological or pathological status of CNS and makes CSF an attractive source of potential biomarkers for neurologic diseases.

Abundances of several CSF proteins have been approved as diagnostic or prognostic markers in clinical practice. For instance, total tau protein concentration, phospho-tau concentration and presence of 42 amino acid form of β-amyloid are indicative of Alzheimer´s disease [1]. Similarly, the appearance of oligoclonal immunoglobulin bands on electrophoretic gels of a CSF sample suggest the presence of multiple sclerosis [2]. However, for most diseases of the CNS, there are currently no reliable biomarkers with diagnostic, prognostic or therapeutic significance. New biomarkers for neurological diseases are therefore desirable.

CSF is formed partly (~ 20%) by active secretion of ependymal cells in the choroid plexuses of the brain ventricles but mostly by ultrafiltration of blood plasma by choroidal capillaries. CSF composition thus inevitably reflects the composition of blood plasma. However, the total protein concentration in CSF is 50–100 times lower, compared to plasma. While typical plasma protein concentration ranges roughly between 60 and 70 mg/ml, normal CSF protein concentration is 0.2–0.7 mg/ml [3]. Proteomic analysis of CSF is, similarly to blood plasma, complicated by the complexity of its protein composition, by a very high dynamic range of concentrations of individual proteins and, most importantly, by the presence of several highly abundant proteins [4]. The ten most abundant CSF proteins (albumin, IgG, transthyretin, transferrin, α1-antitrypsin, apolipoprotein A, alpha-1-acid glycoprotein, haptoglobin, α2-macroglobulin, complement C3) are the same as in blood plasma and account for more than 80% of total CSF protein concentration. In CSF albumin alone accounts for 60% of total protein exceeding its relative abundance in plasma. On the other side, the relative abundance of immunoglobulins, namely IgG and IgM is significantly lower in CSF compared to plasma [5, 6]. Furthermore, CSF contains specific high abundance proteins, for example, cystatin C and prostaglandin D2 synthase, both being synthesized in CNS [7].

High concentrations of the major proteins limit the depth of CSF proteomic analysis and at least partial removal of the most abundant proteins before LC–MS/MS is needed to increase the coverage of CSF proteome [8]. Since there is no specific method for dealing with the abundant proteins in CSF, researchers have to resort to one of the methods developed for blood serum/plasma. Affinity depletion of the most abundant proteins and relative enrichment of medium and low abundance proteins using combinatorial peptide ligand library are among the most common.

Affinity depletion is based on the specific capture of target proteins using immobilized antibodies or other molecules (protein A/G, Cibacron Blue) with high affinity and specificity for the targets. Simultaneous removal of multiple high abundant proteins has become the major pre-analytical strategy for proteomic analysis of blood plasma [9]. Among the most used affinity depletion systems belong Multiple Affinity Removal Systems (MARS, Agilent) for depleting 2, 6, 7 or 14 most abundant proteins [10, 11], Seppro-IgY-14 (Sigma-Aldrich) and ProteoPrep 20 (Sigma-Aldrich) [12, 13] which remove 14 and 20 most abundant plasma proteins, respectively.

An alternative approach to the affinity depletion is the strategy of relative enrichment of low and medium abundance proteins based on their interactions with an immobilized combinatorial peptide ligand library, known under its market name ProteoMiner (Bio-Rad) [14]. Here, proteins from a complex sample bind to a vast spectrum of immobilized hexapeptide ligands through various types of interactions (ionic, hydrophobic interactions, hydrogen bonding, and van der Waal´s force) with different affinities. The binding partners for each protein are present in limited numbers in the library; high-abundance proteins thus exceed the concentration of their binders, and their excess copies are washed off. Low-abundance proteins do not saturate their ligands and become relatively concentrated on the beads [15]. This approach is expected to enrich medium- and low-abundance proteins while relatively depleting or diluting the highly-abundant ones.

Both strategies, affinity depletion [12, 13] and relative enrichment by a ligand library [16, 17] have been shown to significantly increase CSF proteome coverage, but their relative effect has never been directly compared in a single study. Here, we evaluate the relative benefit of these two pre-analytical strategies side-by-side. Specifically, we evaluate the effect of Multiple Affinity Removal System for depletion of 14 most abundant proteins (MARS 14) and ProteoMiner ligand library on the number of proteins identified in human CSF using a standard LC–MS/MS setup. Since the ultimate goal of both strategies is relative depletion of highly abundant proteins, we will use the term “depletion” for both throughout the manuscript.

## Materials and methods

### Materials

Acetonitrile (ACN) and ammonium bicarbonate (AMBIC) were purchased from Fluka (New Jersey, USA), Dulbecco’s phosphate buffered saline (DPBS), sodium chloride, trifluoroacetic acid (TFA) and urea were from Sigma-Aldrich (St. Louis, USA), Bradford protein assay and iodoacetamide (IAA) were from Bio-Rad (Hercules, USA), dithiothreitol (DTT) and sequencing grade modified trypsin were purchased from Promega (Fitchburg, USA). Centrifugal filters 30 kDa (spin concentrators) and water (Milli-Q) for all experiments were from Merck Millipore (Burlington, USA). Opti-Trap, desalting cartridge (C18) was purchased from Optimize Technologies (Oregon City, USA). Cellulose acetate filters 0.22 µm and 5 kDa MWCO (molecular weight cut-off) spin concentrator were purchased from Agilent Technologies (Santa Clara, USA).

### CSF collection

The CSF samples were collected by lumbar puncture during spinal anesthetics procedures in patients of the Urology Clinic of General University Hospital undergoing minor surgery. The study was approved by the Ethics Committee of General University Hospital, Prague. CSF samples were centrifuged at 1500×*g* for 10 min at 4 °C within 30 min to remove cellular debris. Samples were then ultracentrifuged (Beckman Coulter Optima LE-80 K Ultracentrifuge, rotor SW 28, 120,000×*g*, 2 h, 4 °C) to remove all membranous vesicles and other particles. Obtained supernatants were aliquoted and stored at − 80 °C until analysis.

### Preparation of pooled CSF sample

Individual CSF samples were thawed on ice and protein concentration was determined using Bradford protein assay (Bio-Rad) at 595 nm. A pooled CSF sample was generated from 5 patient samples. Each patient’s sample contributed to the pool the same amount of protein. The pooled CSF sample was divided into aliquots, each representing 500 µg of total protein. These aliquots were used throughout the study, always in technical triplicates.

### Multiple affinity depletion using MARS 14 cartridge

Samples were processed in triplicates, according to manufacturer´s instructions. MARS 14 (Agilent Technologies, Santa Clara, USA) cartridge was tempered at room temperature for 20 min. Then the cartridge was equilibrated with 4 ml of Buffer A. CSF sample (500 µg) was diluted 1:1 in Buffer A and was filtered through 0.22 µm filter (10,000×*g*, 10 min, 4 °C). Since the volume capacity of the spin cartridge is limited the diluted CSF sample was loaded on the MARS 14 cartridge sequentially in several steps. After each centrifugation (100×*g*, 1 min) depleted CSF (flow through) was collected. The cartridge was then washed with 2 × 400 µl of Buffer A. Both washes were combined with the depleted CSF and concentrated using 5 kDa MWCO spin concentrator to 200 µl. Proteins bound to the cartridge were eluted by 2.5 ml of Buffer B as the “waste” fraction and stored at − 80 °C until analysis.

### Relative protein enrichment using ProteoMiner ligand library

Protein enrichment was performed using Protein enrichment small-capacity kit (Bio-Rad, CA, USA) according to the manufacturer’s instructions appropriately adapted to the low concentration of CSF. The kit is designed for 10 mg of proteins. As our CSF samples contained 500 µg of proteins, we used 25 µl aliquots of beads for each CSF replicate. Beads were centrifuged (1000×*g*, 1 min, room temperature) to remove the storage liquid and then washed twice with 200 µl of wash buffer. CSF sample containing 500 µg of proteins was added to the beads and incubated for 2 h at room temperature in a 3D rotation mixer (RH-18, Hangzhou Miu Instruments). The sample was then centrifuged (1000×*g*, 1 min, room temperature) for 1 min, and the flow-through fraction containing the unbound waste was collected. The beads with enriched proteins were washed twice with 200 µl of wash buffer and once with 200 µl of MiliQ water after 5 min incubation on the 3D rotation mixer, all three washes were joined with the waste fraction. Bound proteins were eluted from the beads in three steps each consisting of 15 min incubation in 20 µl of elution reagent and centrifugation. The three eluate samples were combined representing the depleted CSF.

### Reduction, alkylation, digestion, and desalting

Triplicates of CSF samples processed by both workflows and triplicates of crude CSF (representing 100 µg of total protein each) were transferred to 30 kDa cut off filters. The subsequent reduction, alkylation, and digestion of the samples were performed using the FASP (filter-aided sample preparation) protocol [18]. Samples were alkylated for 20 min at room temperature in the dark by 100 µl of iodoacetamide solution (50 mM iodoacetamide in 8 M urea, 0.1 M Tris/HCl pH 8.5). Samples were then washed twice with 50 mM ammonium bicarbonate, and proteomics grade trypsin was added. The samples were digested overnight at 37 °C and peptides were collected by centrifugation. The peptides were desalted on OptiPrep C-18 manually operated cartridge. The cartridge was equilibrated by 700 µl of 80% ACN in 0.1% TFA followed by 600 µl of 0.1% TFA. The sample was loaded on the desalting cartridge and washed with 500 µl of 0.1% TFA. Peptides were eluted by 200 µl of 80% ACN in 0.1% TFA, dried in a speedvac (Eppendorf, Concentrator Plus) and stored at − 80 °C.

### nLC–MS^2^ analysis

LC–MS/MS analyses were performed on a Thermo Orbitrap Fusion (Q-OT-qIT, Thermo) mass spectrometer equipped with Dionex Ultimate 3000 chromatograph. Samples were loaded onto the trap column (Acclaim PepMap300, C18, 5 µm, 300 Å Wide Pore, 300 µm × 5 mm, 5 Cartridges) for 4 min at 15 μl/min in 2% acetonitrile in 0.1% TFA. EASY-Spray column, 50 cm × 75 µm ID, PepMap C18, 2 µm particles, 100 Å pore size was used for the separation. Mobile phase A was composed of 2% acetonitrile in 0.1% formic acid. Mobile phase B was composed of 80% acetonitrile in 0.1% formic acid. The gradient was 90 min long at flow-rate 300 nl/min and temperature 55 °C. Mobile phase B increases from 2 to 40% B at 60 min, 90% B at 61 min, hold for 8 min, and 2% B at 70 min, hold for 15 min until the end of the run. Eluting peptide cations were converted to gas-phase ions by electrospray ionization and analyzed on a Thermo Orbitrap Fusion. Survey scans of peptide precursors from 400 to 1600 m/z were performed at 120 K resolution (at 200 m/z) with a 5 × 10^5^ ion count target. Tandem MS was performed by isolation at 1,5 Th with the quadrupole, HCD fragmentation with a normalized collision energy of 30, and rapid scan MS analysis in the ion trap. The MS2 ion count target was set to 10^4^ and the max injection time was 35 ms. Only those precursors with charge state 2–6 were sampled for MS2. The dynamic exclusion duration was set to 45 s with a 10 ppm tolerance around the selected precursor and its isotopes. Monoisotopic precursor selection was turned on. The instrument was run in top speed mode with 2 s cycles.

### Data analysis

The raw data obtained by LC–MS/MS were analyzed using MaxQuant software (version 1.6.0.1). The false discovery rate (FDR) was set to 1% for both, proteins and peptides. The minimum peptide length was set to 7 amino acids. The enzyme was set to trypsin with a maximum of two missed cleavages. Carbamidomethylation of cysteines was set as a fixed modification. N-terminal protein acetylation and methionine oxidation as variable modifications. Main search peptide tolerance was set to 4.5 ppm. MS/MS mass tolerance was set to 0.5 Da.

The Andromeda search engine was used for the MS/MS spectra search against the UniProt Human database (downloaded in March 2018). The “match between runs” feature of MaxQuant was used to transfer identifications to other LC–MS/MS runs based on their masses and retention time (maximum deviation of 0.7 min). The “match between runs” was also used in quantification experiments. Quantifications were performed with the label-free algorithm in MaxQuant. Data analysis was performed using Perseus 1.6.1.3. software.

### Data presentation

All Venn diagrams were created by the web application BioVenn [19].

## Results and discussion

**Fig. 1:**
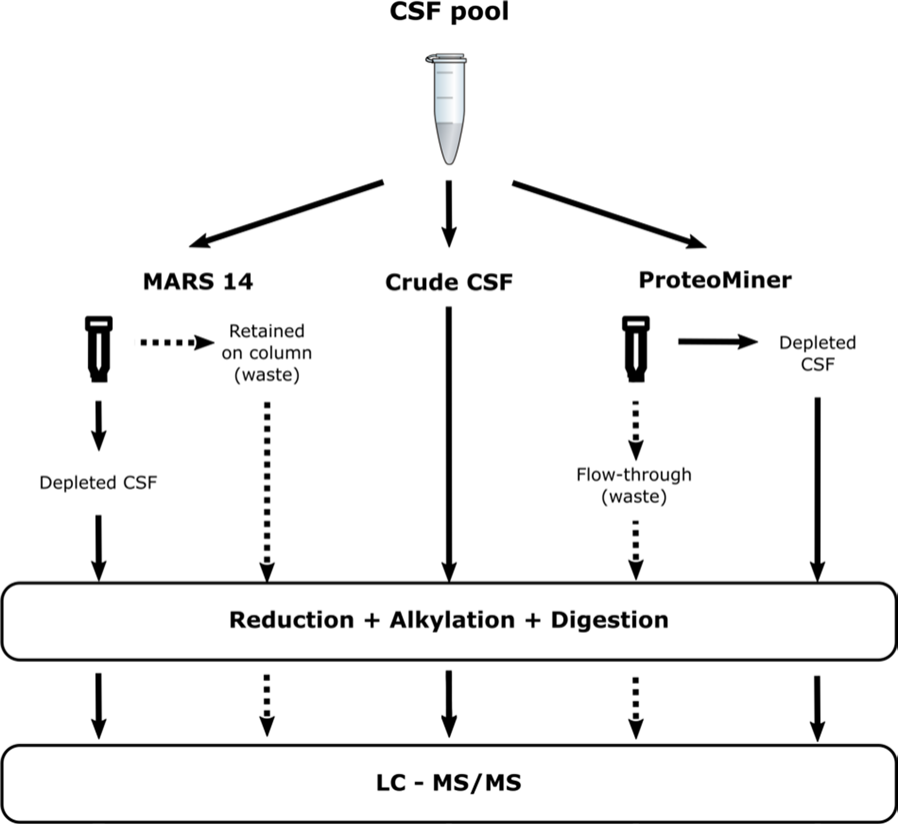
Experimental workflow. Aliquots of a pooled human CSF sample were used. All analyses were performed in triplicates

We evaluated the effect of the two major methods for depletion of abundant plasma proteins in CSF. Specifically, we assessed the impact of MARS 14 cartridge and ProteoMiner immobilized library on the number of proteins identified in triplicate CSF samples by a standard LC–MS/MS. In addition to the key fractions of interest, i.e., depleted CSF samples, we also determined the number of proteins present in the “waste” fractions, which are routinely discarded. (i.e., proteins retained on MARS 14 cartridge and proteins excluded by the ligand library and contained in the ProteoMiner flow-through fraction). All samples originated from a single CSF sample pooled from 5 patients. A non-fractionated crude CSF sample was used as a reference; all samples were processed in technical triplicates. The experimental workflow is depicted in Fig. 1.

All proteins identified in the crude CSF, depleted CSF and corresponding waste fractions are listed in Additional file 1.

### Crude CSF

**Fig. 2:**
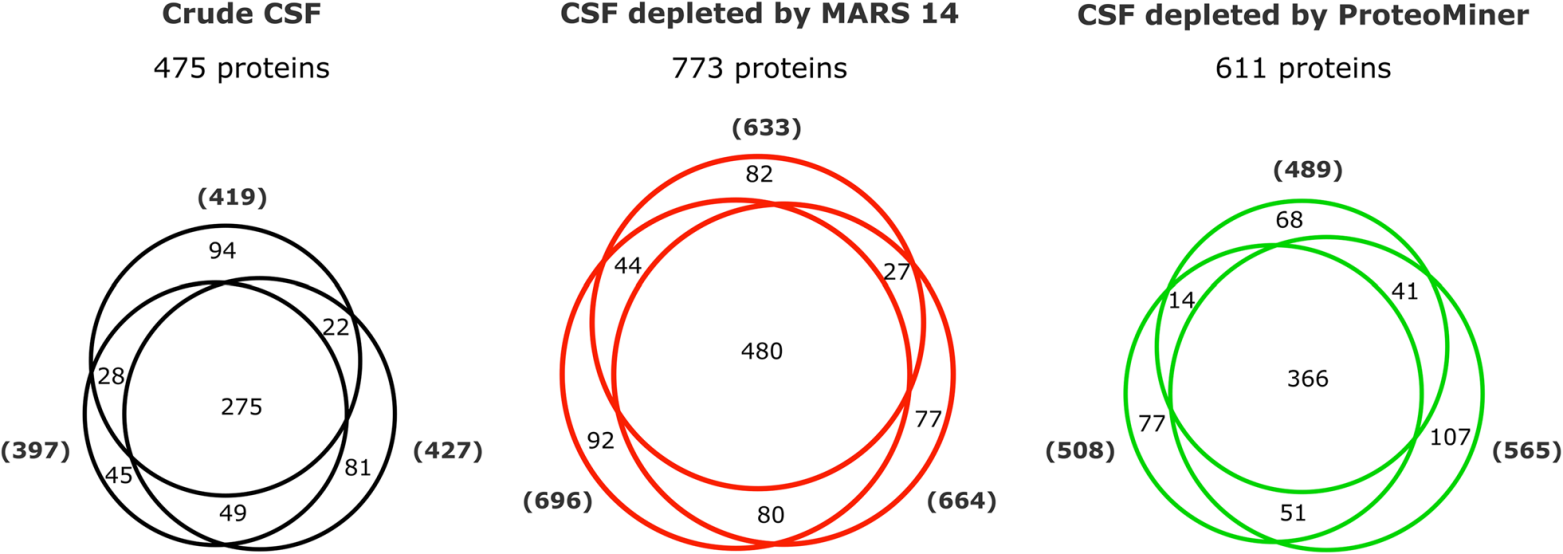
CSF proteome coverage. Numbers of proteins identified by LC–MS/MS in the triplicate analyses of crude CSF, CSF depleted by MARS 14 and CSF depleted by ProteoMiner

LC–MS/MS analysis of crude CSF triplicates provided 397, 419 and 427 identified proteins (Fig. 2). When the MS data of the triplicates were searched together, the analysis resulted in the identification of a total of 475 unique proteins, 275 proteins were present in all three replicates.

The number of identified proteins is with an agreement with a previous study using comparable LC–MS/MS setup [20]. The proteome coverage obtained for crude CSF was used as a reference for evaluation of the effect of CSF processing with MARS 14 cartridge and ProteoMiner library.

### CSF depleted by MARS 14 cartridge

To evaluate the benefit of immunodepletion of major plasma proteins we used Multiple affinity removal cartridge for depletion of the 14 most abundant proteins of human plasma (MARS 14, Agilent), very often used in proteomic biomarker studies. Depletion of the most abundant CSF proteins using MARS 14 cartridge increased the number of identified proteins by 63% compared to crude CSF to total 773 in the triplicate analysis (Fig. 2). We identified between 633 and 696 proteins in the triplicate analyses. Of the total 773 identified proteins, 480 proteins were found in all three replicates.

### CSF depleted with ProteoMiner ligand library

The principally different method of relative protein enrichment of medium and low abundance protein using ProteoMiner hexapeptide ligand library increased the number of identified CSF proteins by 29% compared to crude CSF. The total number of unique proteins identified in the ProteoMiner-treated CSF triplicate was 611 (Fig. 2), 366 proteins were identified across all three replicates.

Considering the number of identified proteins, it is evident, that both depletion strategies are effective and significantly increase the number of identified proteins in comparison to crude CSF. However, MARS 14 immunodepletion enabled identification of a substantially more proteins.

### Depletion efficiency

To evaluate the efficiency of depletion of the most abundant proteins we used label-free quantitative (LFQ) analysis. Relative depletion of the major proteins was calculated from the normalized intensities of peptide signals in the crude CSF and the depleted CSF. Results can be seen in Table 1. The depletion efficiency of MARS 14 cartridge for the 14 most abundant (plasma) proteins was high, comparable with the efficiency claimed by the manufacturer and confirmed by others [10]. In general, CSF depletion with ProteoMiner decreased the amounts of the individual 14 most abundant proteins less effectively.

**Table 1:** The efficiency of depletion of the most abundant 14 plasma proteins by the MARS 14 immunodepletion and ProteoMiner

			MARS 14	ProteoMiner
			%Removed	%Removed
P02768	Serum albumin	ALB	97.4	71.9
P02647	Apolipoprotein A-I	APOA1	99.9	< 33
P02652	Apolipoprotein A-II	APOA2	75.6	< 33
P01876	Ig alpha-1 chain C region	IGHA1	99.9	97.8
P01023	Alpha-2-macroglobulin	A2M	99.9	68.4
P02763	Alpha-1-acid glycoprotein 1	ORM1	99.7	97.8
P01009	Alpha-1-antitrypsin	SERPINA1	99.7	84.3
P01857	Ig gamma-1 chain C region	IGHG1	99.6	70.9
P02787	Serotransferrin	TF	99.4	96.1
P00738	Haptoglobin	HP	98.7	92.1
P01024	Complement C3	C3	98.1	<33
P02766	Transthyretin	TTR	95.2	89.0
P02675	Fibrinogen beta chain	FGB	94.5	< 33
P02671	Fibrinogen alpha chain	FGA	93.7	< 33
P02679	Fibrinogen gamma chain	FGG	91.7	< 33
P01871	IgM	IGHM	100^a^	< 33

Data from label-free quantification based on MS1 peptide signal intensities

^a^IgM was not detected in the CSF after MARS 14 immunodepletion

### The depleted CSF

The two compared strategies employ radically different principles of dealing with the most abundant proteins, i.e., specific interactions with antibodies and other molecules versus less defined interactions with a library of peptide ligands. Also, MARS 14 cartridge retains unwanted proteins bound on the stationary phase, while ProteoMiner excludes the abundant proteins in the mobile phase.

**Fig. 3:**
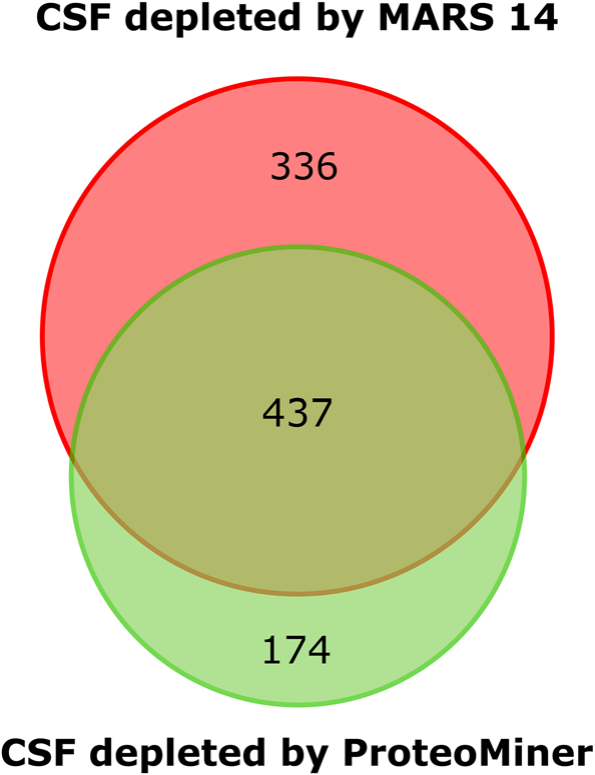
The unique and shared proteins identified in depleted CSF after depletion by MARS 14 or ProteoMiner. Over 330 proteins identified in CSF depleted by MARS 14 cartridge were not found in the CSF depleted by ProteoMiner library. Vice versa, 174 proteins were uniquely identified only in the CSF sample after ProteoMiner depletion

The principal differences in the two depletion strategies and their different depletion efficiencies may affect not only the number of identified CSF proteins but can also result in the identification of different sets of proteins. We compared the lists of proteins identified in CSF depleted by MARS 14 and ProteoMiner and confirmed that both methods provided a large number of unique proteins not identified by the other method (Fig. 3). The depletion of CSF using MARS 14 cartridge provided 336 such proteins not identified in the CSF treated by ProteoMiner and vice versa, the CSF sample treated with ProteoMiner provided 174 unique proteins not observed in the CSF depleted by MARS 14. This suggests that both methods introduce a bias toward and against some particular proteins.

We evaluated three basic properties of the identified proteins, namely MW, pI and hydrophobicity (GRAVY score) of all proteins identified in the depleted CSF samples. There was no systematic preference regarding MW, pI and hydrophobicity (GRAVY score) distributions between the two depleted fractions neither between the depleted samples and crude CSF (Additional file 2).

One example of depletion bias between these two methods is a higher number of various immunoglobulins found in the CSF sample depleted by ProteoMiner (33 identified proteins) compared to CSF treated by MARS 14 (10 proteins). In this case, it can be easily explained by the fact that MARS 14 specifically and efficiently targets and depletes immunoglobulins. However, among the method-specific proteins were also molecules previously identified as specific brain-enriched proteins [21] and therefore molecules with biomarker potential. For example, brain-enriched delta and Notch-like epidermal growth factor-related receptor (DNER_HUMAN), or Myelin-associated glycoprotein (MAG_HUMAN) were present only in the CSF depleted by MARS 14 while Protocadherin-8 (PCDH8_HUMAN) and Protocadherin gamma-C5 (PCDGM_HUMAN) were identified only in the CSF sample after ProteoMiner depletion [21]. In summary, both depletion methods have a profound beneficial impact on CSF proteome coverage. However, the lists of identified proteins differ between the two approaches suggesting a potential loss of biomarker proteins. To further explore the issue we analyzed the waste fractions generated in both workflows.

### The waste fractions

Several studies reported that immunodepletion of high-abundant proteins leads to an undesired co-depletion of numerous non-targeted proteins from the CSF [12, 13], including potential biomarkers of neurologic diseases [22]. Although we found no comparable study addressing the losses of CSF proteins in the ProteoMiner workflow, it can be expected that analogical losses occur, either due to low binding to the hexapeptide library or due to strong interactions with the abundant proteins that oversaturate their binding ligands.

To compare the extent of such unwanted protein losses due to co-depletion in both workflows, we analyzed the fraction of proteins retained by the MARS 14 cartridge and the equivalent proteins present in the flow-through fraction of the ProteoMiner library workflow. These waste fractions are routinely excluded from further analysis and discarded.

**Fig. 4:**
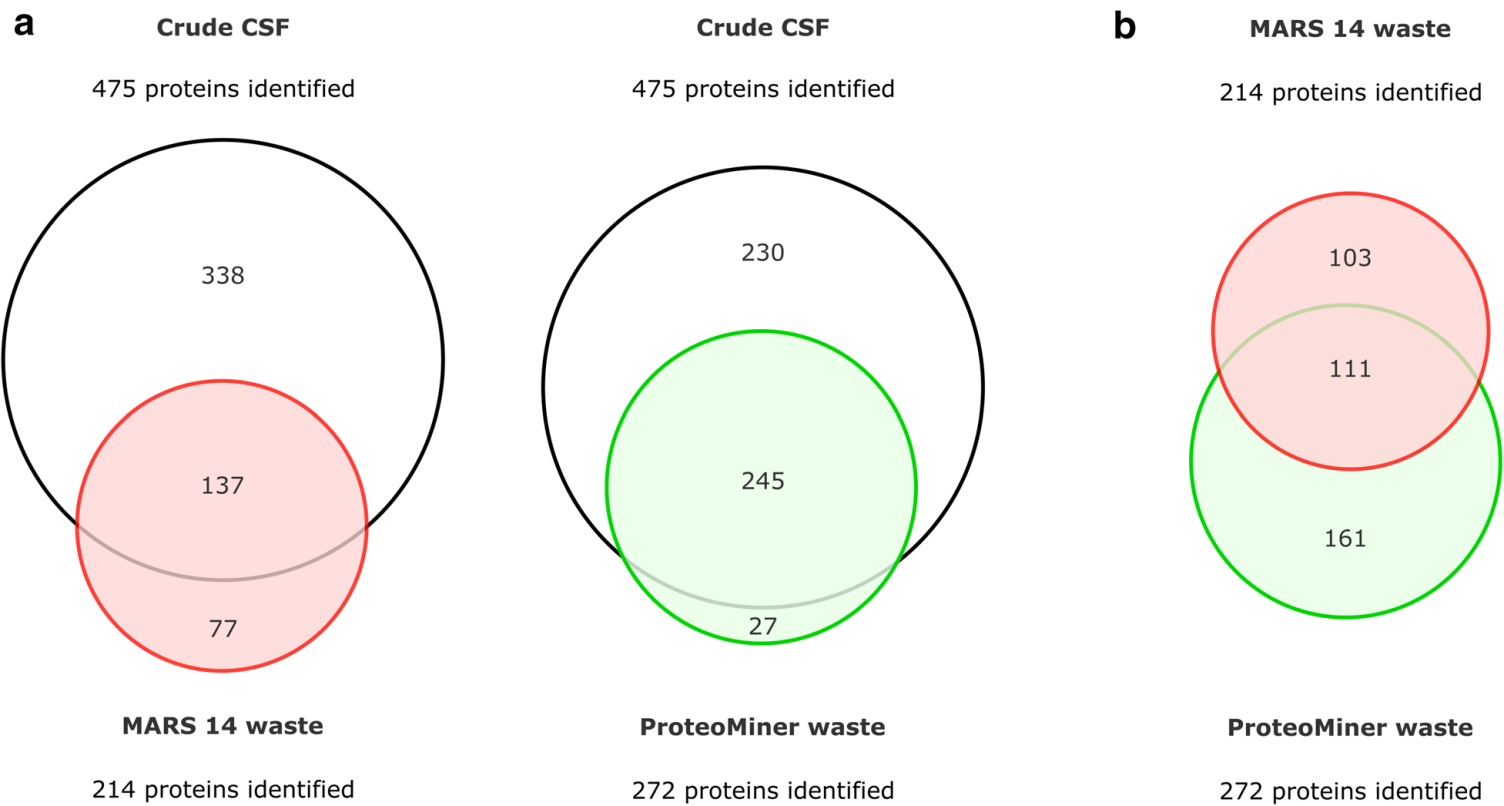
Proteins identified in the waste fractions (proteins retained by MARS 14 cartridge and proteins excluded in the ProteoMiner flow-through). **a** The overlap of the proteins identified in the waste fractions with the proteins found in crude CSF. **b** The overlap between the two waste fractions

LC–MS/MS analysis of the waste fractions revealed significant extent of CSF protein loss in both depletion workflows. In addition to the most abundant CSF proteins (albumin, IgG, transthyretin, transferrin, α-1-antitrypsin, haptoglobin α, etc.), both waste fractions contained a substantial number of co-depleted non-target proteins. MARS 14 cartridge retained 214 proteins, and the ProteoMiner flow-through fraction contained 272 identified proteins (Fig. 4a).

The significant number of the proteins identified in the waste fractions was also identified during the analysis of crude CSF. Large overlap with the crude CSF was found in the ProteoMiner waste (91% identified proteins), while in the MARS 14 waste only 64% of identified proteins were also seen in the crude CSF (Fig. 4a). Although the numbers of the proteins identified in both waste fractions were roughly comparable (214 vs. 272), the lists of the proteins differed significantly, only 111 proteins were present in both fractions. 103 and 161 proteins were specific for MARS 14 and ProteoMiner waste, respectively (Fig. 4b). Both these observations seem to reflect the fundamentally different fractionation principles of the depletion methods. However, again there was no systematic bias regarding MW, pI and hydrophobicity (GRAVY score) distributions between the two waste fractions or compared to depleted or crude CSF samples (see Additional file 2).

**Fig. 5:**
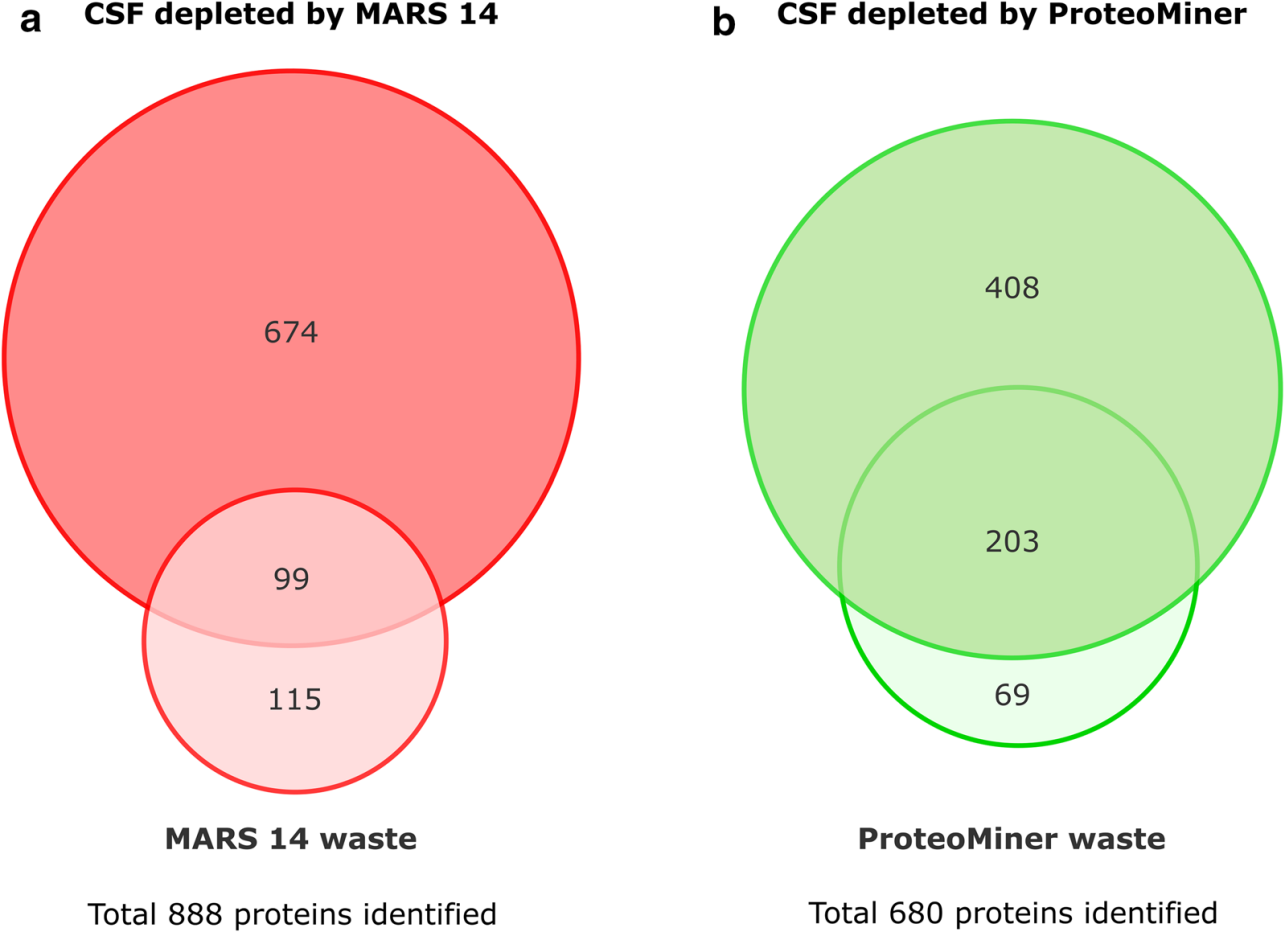
The total number of proteins identified in the depleted CSF and corresponding waste fractions

The high numbers of proteins identified in the waste fractions confirm the alarmingly large extent of the unwanted loss of proteins in both workflows. Importantly, many of the CSF proteins identified in the waste fractions were unique for the waste, i.e., were not found in the corresponding depleted CSF samples. We identified 115 and 69 such proteins in MARS 14 and ProteoMiner waste fractions, respectively (Fig. 5). Addition of the waste-specific proteins to the list of proteins identified in the corresponding depleted CSF increased the total coverage of CSF proteome roughly by 10-15 percent to 888 and 680 identified proteins in MARS 14 and ProteoMiner workflow, respectively (Fig. 5). The strikingly high number of proteins lost during both depletion workflows may advocate future inclusion of the waste fractions into CSF biomarker studies. Such inclusion would be beneficial, especially if the waste fraction contained not only the major plasma constituents but also CNS-specific proteins. To evaluate the presence of such molecules, we compared the lists of proteins identified in the waste fractions with a list of previously characterized as brain-enriched proteins [21]. We found several such molecules to be present in the waste fractions, namely cell adhesion molecule 2 (CADM2_HUMAN), Amyloid-like protein 1 (APLP1_HUMAN) or Beta-Ala-His dipeptidase (CNDP1_HUMAN). Further search in literature identified additional waste-specific proteins with known relevance for neurophysiology and neuropathology. For example, MARS 14 cartridge fully retained Kinesin-like protein KIF1B (KIF1B_HUMAN) linked to Multiple Sclerosis susceptibility [23] and Tubulin beta-3 chain (TBB3_HUMAN) required for axon guidance [24]. Similarly, ProteoMiner waste fraction (but not CSF depleted by ProteoMiner) contained Proenkephalin-A (PENK_HUMAN) which has been considered as a potential marker of dementia and acute neuroinflammatory disorders in the form of its stable fragment (MR-PENK A) [25]. Proenkephalin-A was also described as an indicator of severity and clinical outcome in patients with ischemic stroke [26].

## Conclusions

CSF is a valuable source of information on the status of CNS and a potential treasure trove of biomarkers. Compared to blood plasma, proteomic analysis of CSF is limited by its low protein concentration and the maximum volume of CSF that can be safely taken during the lumbar puncture. Considering also the potential health risks of the lumbar puncture, it is imperative to make proteomic analyses of CSF as efficient as possible. To maximize the number of identified CSF proteins we compared the benefits of two frequent, but principally different, methods for relative depletion of major plasma proteins—MARS 14 cartridge and ProteoMiner ligand library. To our knowledge, this is the first side-by-side comparison of these two major strategies in CSF. In our hands, both methods markedly improved CSF proteome coverage compared to analysis of crude (non-depleted) CSF. However, both methods enabled identification of exclusive sets of proteins in the resulting depleted CSF, reflecting the distinct principles of the fractionation.

The obvious beneficial effect of major protein depletion, however, comes for the price of unwanted protein loss during the procedure. Depletion or removal of any protein from a complex protein mixture under non-denaturing conditions inevitably leads to a co-depletion of other, non-targeted, proteins. In the case of MARS 14, it is either due to their direct interaction with the targeted proteins, due to cross-reactivity or a nonspecific interaction with the antibody, or via interactions with the stationary phase matrix. In the case of ProteoMiner, it may be due to insufficient interaction of proteins with the hexapeptide library or due to strong interactions with the excluded highly abundant proteins. In both tested methods, we observed a significant number of unique co-depleted proteins (proteins not identified in the corresponding depleted CSF samples), including brain-enriched proteins, proteins with potential or confirmed roles in CNS pathologies—i.e., potential biomarkers. This clearly showed, that to maximize CSF proteome coverage and to limit the potential loss of biomarkers during the depletion, proteins retained by the MARS 14 cartridge (or excluded by ProteoMiner beads) should also be analyzed in future CSF biomarker studies. Alternatively, the addition of a crude CSF sample analysis may partially avoid the loss of some proteins due to co-depletion as the proteins contained in the waste fractions are largely a subset of crude CSF. However, in contrast to the waste fraction, the inclusion of crude CSF would require an additional volume of CSF.

Nevertheless, our main aim was to determine which of the two depletion methods enables higher coverage of the CSF proteome in an identical LC–MS/MS setup. MARS 14 cartridge enabled markedly higher proteome coverage with 773 identified proteins compared to ProteoMiner (611) and crude CSF (475). The superior performance of the MARS 14 cartridge can be further emphasized by quicker sample processing. Additionally, depletion of a single CSF sample using MARS 14 cartridge is less expensive, despite higher acquisition cost of MARS 14 cartridge.

To further increase the CSF coverage and make biomarker discovery more successful, novel strategies may be needed (in addition to a more extensive peptide fractionation). Among such tactics may be an orientation to specific forms of information carried by the CSF, namely neuropeptides or extracellular vesicles. These potential information carriers are usually excluded or lost in the standard proteomic analyses of CSF because of their low MW or sedimentation, respectively. Extracellular vesicles are cell-derived membrane structures carrying tissue-specific information and play a role in intercellular communication [27] and can be isolated from patient CSF and analyzed by LC–MS/MS [28]. Similarly, endogenous (neuro)peptides can be released from their carrier proteins, collected by ultrafiltration and analyzed [29]. Such semi-targeted approaches may reveal a brand new level of information carried by CSF.

## Additional files

**Additional file 1.** A list of all identified proteins including crude CSF, depleted CSF and the waste fractions.

**Additional file 2.** Distributions of MW, pI and hydrophobicity (GRAVY score) in crude CSF, depleted CSF and the waste fractions.

## Abbreviations

ACN: acetonitrile
AMBIC: ammonium bicarbonate
CNS: central nervous system
CSF: cerebrospinal fluid
DTT: dithiothreitol
IAA: iodoacetamide
LC–MS/MS: liquid chromatography—tandem mass spectrometry
MARS: multiple affinity removal system
MW: molecular weight
pI: isoelectric point
TFA: trifluoracetic acid
