# Supplementary material for: Affinity depletion versus relative protein enrichment: a side-by-side comparison of two major strategies for increasing human cerebrospinal fluid proteome coverage
Source: Clin Proteomics. 2019 Feb 26;16:9. doi: 10.1186/s12014-019-9229-1 (PMC6390343; doi:10.1186/s12014-019-9229-1)
Supplement: Supplementary file 2 — Additional file 2. Distributions of MW, pI and hydrophobicity (GRAVY score) in crude CSF, depleted CSF and the waste fractions. [file 12014_2019_9229_MOESM2_ESM.pdf]

Molecular weight distributions

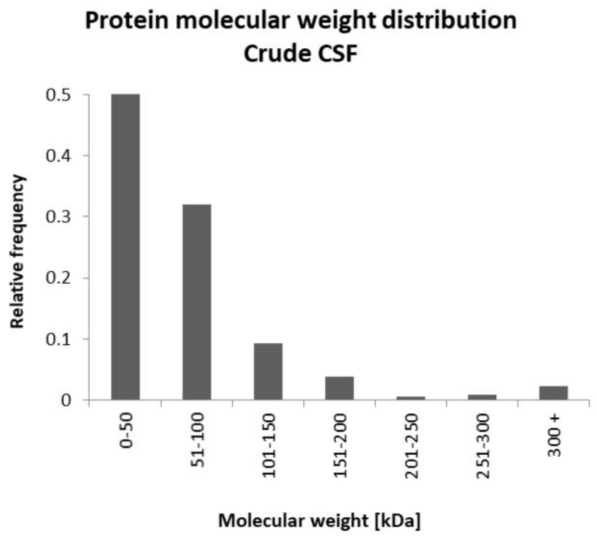

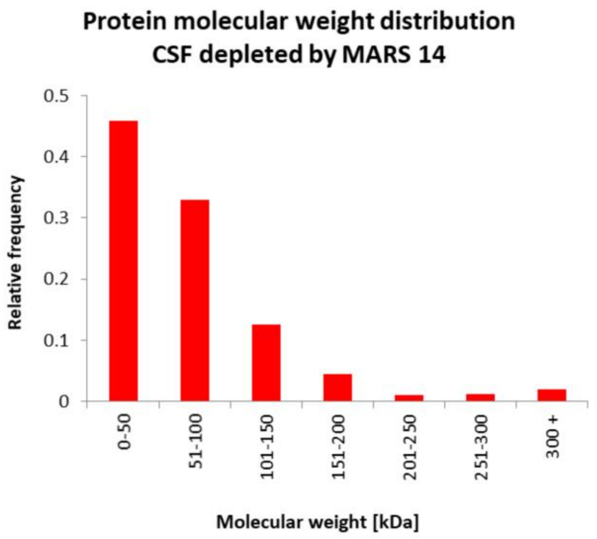

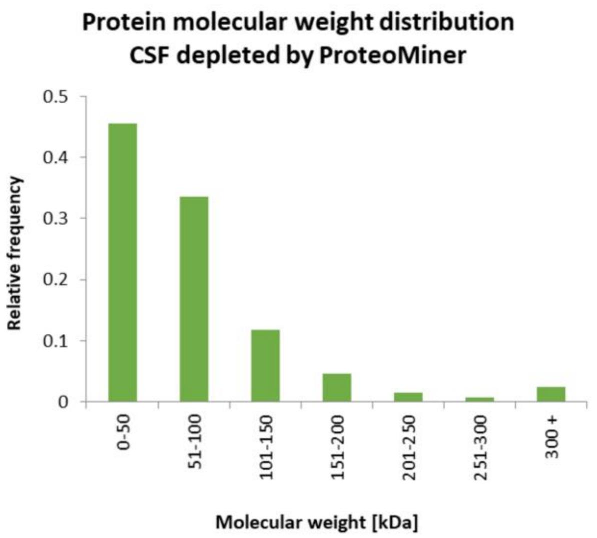

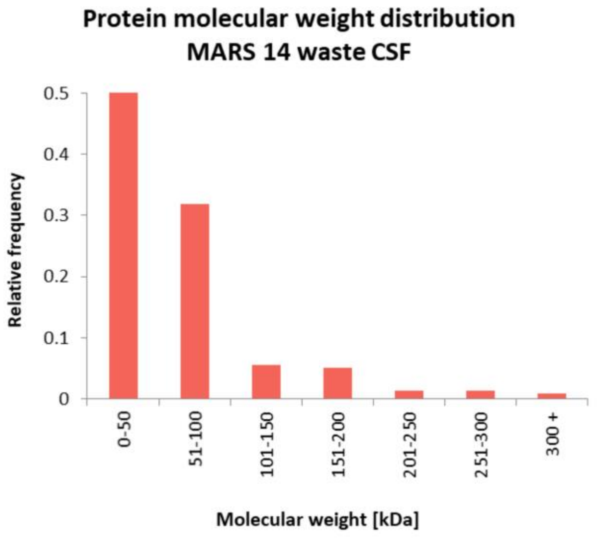

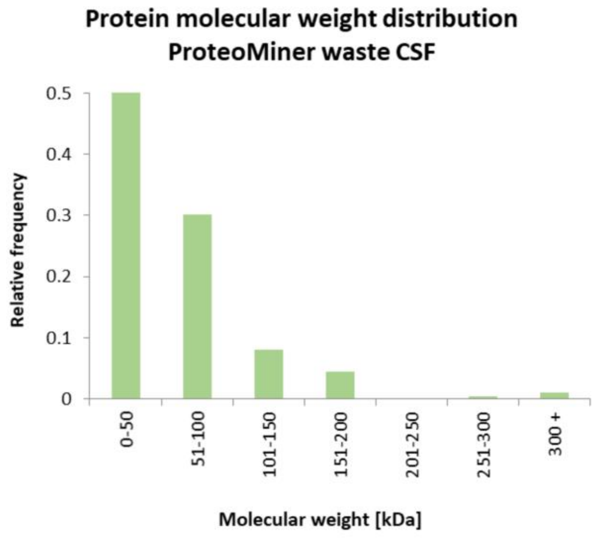

Isoelectric point distributions

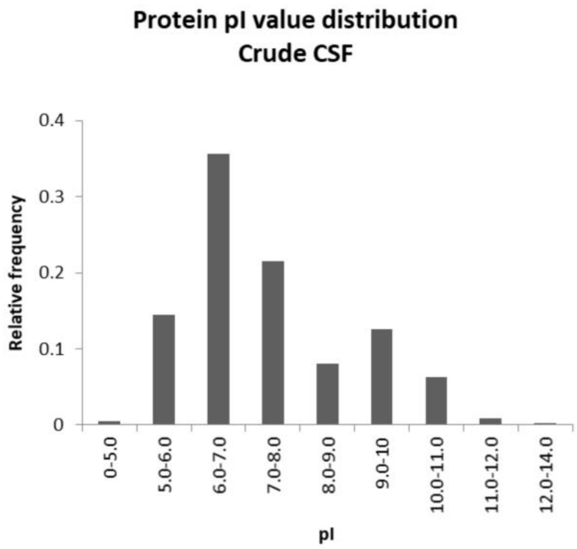

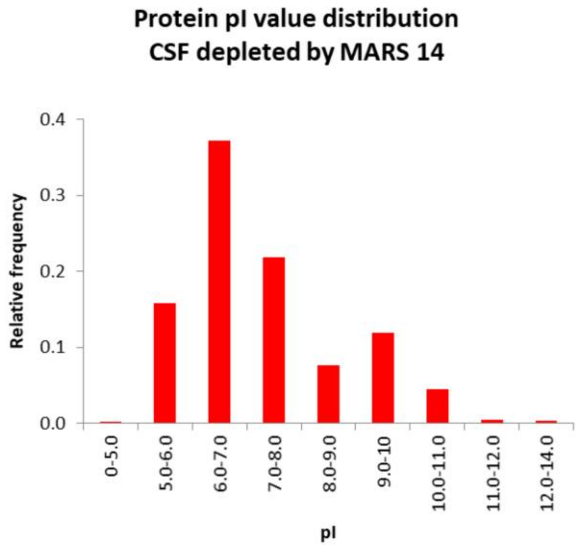

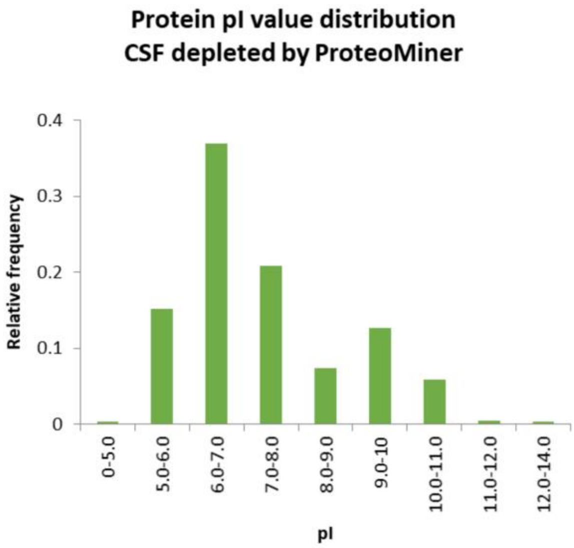

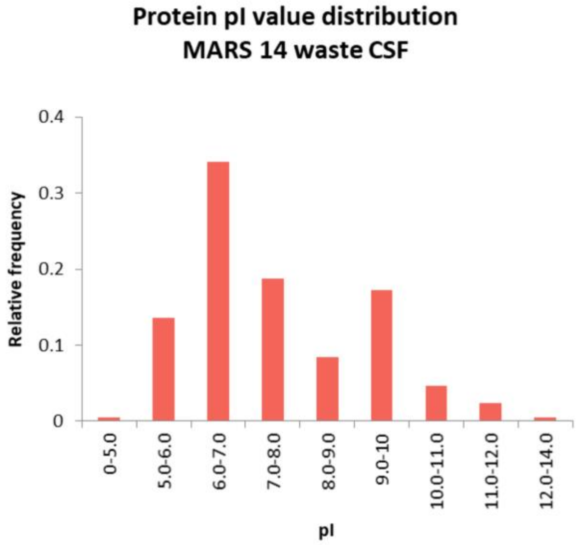

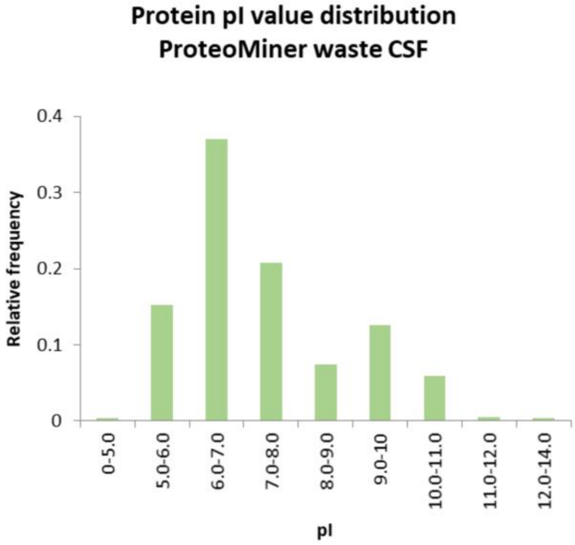

Hydrophobicity (GRAVY score distributions)

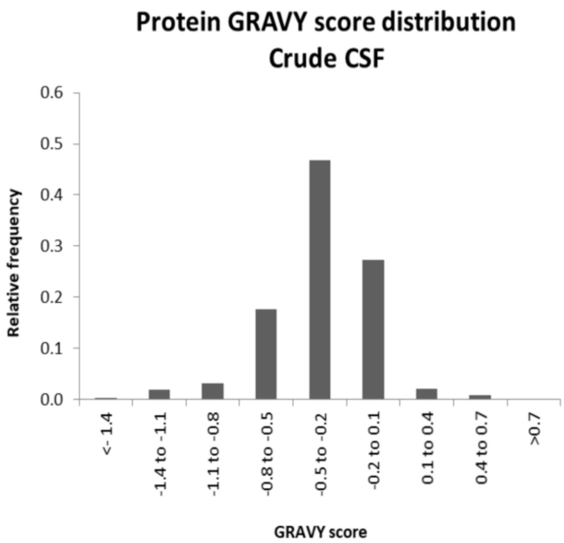

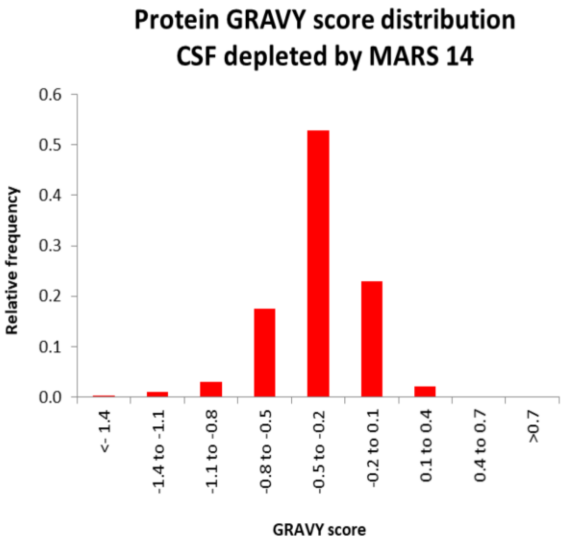

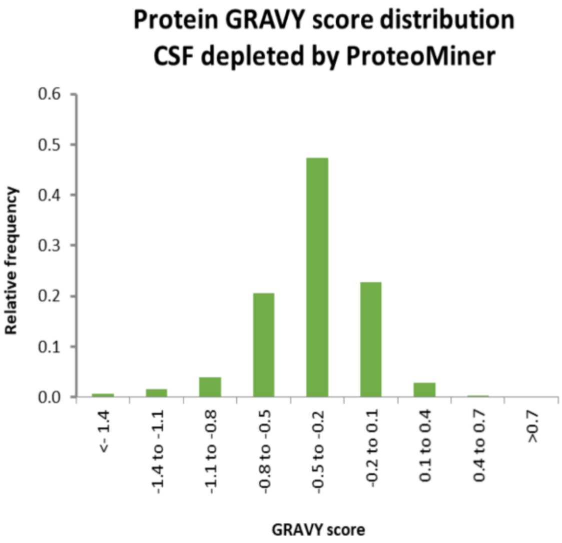

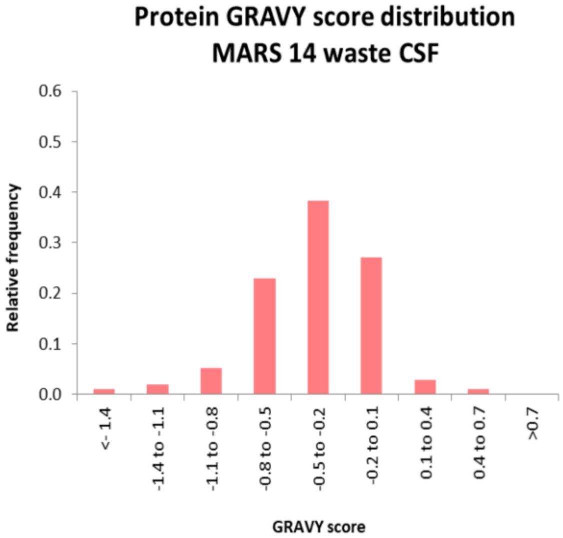

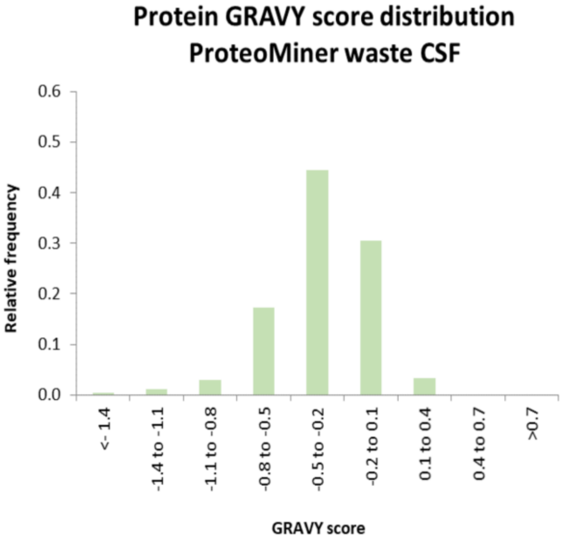

Gravy scores were calculated using http://www.gravy-calculator.de/

J. Kyte, R. F. Doolittle, "A simple method for displaying the hydropathic character of a protein". *J. Mol. Biol.*, vol. 157, no. 1, pp. 105–132, 1982; doi:10.1016/0022-2836(82)90515-0
